# The use of long-acting Aripiprazole in a multi-center, prospective, uncontrolled, open-label, cohort study in Germany: a report on global assessment of functioning and the WHO wellbeing index

**DOI:** 10.1186/s12888-020-02488-1

**Published:** 2020-02-22

**Authors:** Daniel Schöttle, Wolfgang Janetzky, Daniel Luedecke, Elmar Beck, Christoph U. Correll, Klaus Wiedemann

**Affiliations:** 1 Klinik für Psychiatrie und Psychotherapie, Zentrum für Psychosoziale Medizin, Universitätsklinikum Hamburg-Eppendorf, Martinistrasse 52, 20246 Hamburg, Germany; 2 Lundbeck GmbH, Ericusspitze 2, 20457 Hamburg, Germany; 3 ANFOMED GmbH, Röttenbacher Str. 17, 91096 Möhrendorf, Hamburg, Germany; 4 The Zucker Hillside Hospital, Department of Psychiatry, Hofstra Northwell School of Medicine, Northwell Health, 75-59 263rd St, Glen Oaks, NY 11004 USA; 5 Department of Psychiatry and Molecular Medicine, Charité Universitätsmedizin, 500 Hofstra Blvd, Hempstead, NY 11549 USA; 6 Department of Child and Adolescent Psychiatry, Augustenburger Platz 1 (Mittelallee 5A), 13353 Berlin, Germany

**Keywords:** Long-acting injectable, LAI, Naturalistic, Schizophrenia, Schizoaffective, Patient perspective

## Abstract

**Background:**

In this non-interventional study, the functionality and well-being of patients with schizophrenia with aripiprazole once-monthly (AOM) was evaluated under real-life conditions in a naturalistic population.

**Methods:**

This non-interventional, prospective, multicenter 6-month study included 242 predominantly symptomatically stable patients (mean age 43.1 ± 15.1 years, 55% male) who switched their treatment to AOM after 9.7 (± 22.3) months of oral treatment. Outcome parameters included functionality (Global Assessment of Functioning, GAF), patient’s wellbeing (WHO-5 Well-Being Index, WHO-5), and both patient’s and clinician’s assessment of efficacy and tolerability of AOM. Treatment emergent adverse events (TRAE) were also recorded.

**Results:**

At baseline, the mean GAF score was 47.0 (±13.9), indicating that patients experienced serious impairment in functioning. A continuous increase to 60.2 (±17.0) during treatment was found, with a robust and significant increase already after 4 weeks. At study start, patients reported diminished wellbeing, with a mean score of 10.6 (±5.6) on the WHO-5 scale. During treatment, patient wellbeing increased continuously with strong and significant improvements even after 4 weeks and an overall improvement of 4.8 (±6.9) over the course of 6 months with an endpoint of 15.4 (±5.5). Stratification of these results showed that more pronounced effects were achieved in younger patients ≤35 years (p<0.05 for GAF). The effectiveness and tolerability of AOM was rated good/very good by most patients (89.2 and 93.7%) and physicians (91.4 and 96.8%). Only few TRAEs occurred.

**Conclusions:**

Our results show a significant positive effect after initiation of AOM treatment in predominantly stable patients with schizophrenia on their functioning and wellbeing, which was even more pronounced in patients aged ≤35 years, thereby supporting previous randomized controlled findings under routine conditions in clinical practice.

## Background

Schizophrenia is a mental disorder starting in adolescence, that severely impairs patients` functioning and diminishes their quality of life as well as their general wellbeing [1]. In general, schizophrenia is characterized by recurring episodes characterized by positive symptoms, such as hallucinations and thought disorders, as well as negative symptoms, like blunted affect and avolition. Treatment of these negative symptoms is challenging, as they may persist even after remission from an acute episode and as they can have a further negative impact on quality of life and general wellbeing. Therefore, patients may not regain their previous level of functioning and quality of life [2, 3]. For this reason, it is highly important to intervene early after onset of the disease to prevent relapses and restore functional, cognitive, and affective capabilities [4]. For relapse prevention, adherence to pharmacological and psychosocial treatment is critical. However, independent of having a first or having had multiple psychotic episodes, nonadherence rates in patients with schizophrenia are high [5], and the reasons for nonadherence are complex [6–8]. Adherence to pharmacological treatment may be improved when long-acting injectables (LAIs) are used, which has been mainly demonstrated under real life conditions [9–14]. Aripiprazole once-monthly (AOM) is an atypical LAI that was shown to elicit clinically relevant and lasting improvements in the patients` quality of life in the QUALIFY study [15, 16], accompanied by improvements in functioning and ability to work [17]. Furthermore, pre-specified exploratory analyses of age groups showed that younger patients (≤35 years of age) benefited even more from the treatment [15]. Furthermore, patient functioning and quality of life were superior during AOM treatment compared with oral standard-of-care in a naturalistic study [18].

The present non-interventional study in a naturalistic setting under routine treatment conditions with a heterogenous patient sample was designed to confirm the results of the previous randomized controlled clinical studies [19–21], which were conducted in a more homogenous population of patients. As the physician’s view on the severity of the disease and improvement of psychosocial functioning and quality of life may differ from the patients` perspective [22, 23], it is important to assess both perspectives by collecting information from both groups. How patients experience a beneficial effect not only regarding symptomatic improvement and low rates of side-effects, but also how they evaluate subjectively important domains, such as psychosocial functioning and their general quality of life, becomes ever more important with increasing therapy duration as it impacts the will to adhere to and continue with the treatment [24–26].

In a previous study in the same patient cohort, effectiveness measurements of psychopathology (Brief Psychiatric Rating Scale, BPRS) and severity of illness scales (Clinical Global Impression–Severity, CGI-S and Clinical Global Impression–Improvement, CGI-I), were analyzed and discussed [27]. Briefly, at baseline, most of the patients were markedly ill and had predominantly stable symptomatology for an average of 5.9 months (SD: 18.2), with 91 patients (39.2%) being stable for < 1 month and 28 (12.1%) not being stable at all. They had a mean global BPRS value of 54.1 (SD 15.6), and a mean CGI-S value of 4.8 (SD 0.8). The reduction in global BPRS was − 13.8 (SD: 16.0; *p* <  0.001) at follow-up. The proportion of patients with high (worse) CGI-S scores decreased (p <  0.001), and the proportion of patients with low scores increased significantly (*p* < 0.001). All together, 35.3% of the patients improved by one grade on the CGI-S score (in Fig. 6 of the original publication [27], a portion of 38.3% was mistakenly given); 24.3% of the patients improved by 2 grades or even more.

In this multicenter, prospective, non-interventional study, 242 patients with schizophrenia were treated with AOM and monitored over the course of 6 months. The treating clinicians were asked to estimate the patient’s level of functioning on the Global Assessment of Functioning (GAF) scale. Patient wellbeing was assessed using the WHO-5 wellbeing index. Efficacy and tolerability of AOM was rated by the patients as well as the clinicians. Thus, we hypothesized that 6 months of AOM treatment would significantly improve functional status and wellbeing of patients receiving usual-care based AOM treatment.

## Methods

### Design

This multicenter, prospective, 6-month, uncontrolled, open-label, cohort study was designed and conducted in a naturalistic setting according to the German Medicinal Product Act and approved by the Freiburg ethics commission international (Approval number: 014/1336).

Data were collected from 75 German centers, including outpatient clinics and resident physicians, between July 2014 and March 2016. The choice of treatment with AOM was independent from the inclusion in the study cohort.

**Fig. 1:**
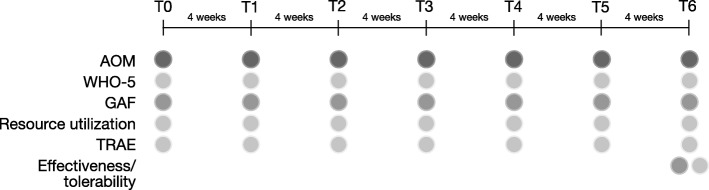
Study design. Patients were treated with AOM at seven time points (T0-T6) in four-week intervals. Data was collected at the time points indicated. Light gray color indicates statements by the patient, dark gray color statements by the physician. AOM, Aripiprazole once-monthly; WHO-5, WHO-5 wellbeing index; GAF, Global Assessment of Functioning; TRAE, treatment related adverse events

Patients were seen about every 4 weeks (− 2/+ 5 days) at seven time points during the study (T0-T6, Fig. 1). Data were collected using patient and clinician questionnaires at each time point.

### Patients

Inclusion criteria for patients were: age ≥ 18 years, diagnosis of schizophrenia (F20.X) according to ICD-10, and treatment with AOM according to prescribing information on an outpatient basis. The choice to switch from oral aripiprazole to treatment with AOM was made prior to inclusion in the cohort. Patients gave written consent to participate.

Exclusion criteria were contraindication to AOM, being a member or being related to a member of the study staff, pregnancy, planning a pregnancy, nursing, or probable reluctance to adhere to the monitoring plan (evaluated by the treating physician).

Patients were regarded as clinically stable when having no pronounced fluctuations in their symptoms and when taking their oral medication at a stable dosage for several weeks. Being stable was not clearly defined, but decided by the prescribing clinician on an individual basis.

### Assessments

In the Global Assessment of Functioning (GAF) scale, physicians are asked to give the patient’s overall functioning as a single value between 100 (excellent functionality) and 1 (persistent danger of severely hurting self or others *or* persistent inability to maintain minimal personal hygiene *or* serious suicidal act with clear expectation of death). Possibly clinically relevant changes were defined as 4, 10 or 12 points, according to Amri et al. [28].

The WHO-5 wellbeing index is a self-rating short questionnaire comprising five items to assess a patient’s wellbeing, balancing the wanted and unwanted effects of treatments [29]. It contains statements about the patient’s wellbeing, and the patients` estimate of the amount of time this was true during the last 2 weeks between 0 (at no time) and 5 (all of the time), yielding a total score of 0–25. A score of ≤13 is considered indicative of being at risk for depression [29]. Patients completed this questionnaire at every other visit (T0-T6). A change of ≥10% (here corresponding to 2.5 points) is considered clinically relevant [29].

The effectiveness and tolerability of AOM treatment was rated by both clinicians and patients on a four-point Likert scale ranging from “very good”, “good”, “moderate” to “poor”.

Adverse events were reported by the patients at every other visit and categorized by the treating clinicians as treatment-related (TRAE) or unrelated events.

Other rating scales also used in the same study, but not presented here, are discussed elsewhere [27].

### Statistical analysis

Due to the non-interventional design of the study, all data were collected as descriptive statistical values. Missing values were complemented by the Last Observation Carried Forward (LOCF) method if there was a value for T0 and at least one other time point. Data were processed using SAS™ software. No statistical hypotheses were formulated, and statistical tests were exploratory. The Wilcoxon Signed-Rank test was used for paired samples, and the Wilcoxon’s rank-sum test for independent samples. Changes in marginal distributions in contingency tables of categorical outcomes were analyzed using Bhapkar’s test [30], and proportions within one group of patients were analyzed with the binomial test. Fisher’s exact test was used to compare proportions between groups of patients. Subgroup comparisons by age group were covaried using a linear regression analysis with backward selection of effects for variables that differed significantly between the two age groups at baseline at *p* < 0.05 (see Table 1). All tests were two-sided with alpha = 0.05, without correction for multiple testing.

**Table 1:** Patient baseline demographics

	All patients (*n* = 242)	Patients ≤35 years (*n* = 89)	Patients > 35 years (*n* = 153)	*p* value for comparison of age groups
Age, years (SD)	43.1 (15.1)	28.8 (4.3)	51.3 (12.7)	< 0.0001^a^
Sex, male, n (%)	133 (55.0)	57 (64.0)	76 (49.7)	0.0328^b^
Family status, married or in a relationship, n (%)	53 (22.0)	13 (14.8)	40 (26.1)	0.0520^b^
Employment status, n (%)				< 0.0001^b^
Employed	43 (18.0)	23 (26.4)	20 (13.2)	
Unemployed	73 (30.5)	36 (41.4)	37 (24.3)	
Annuitant	99 (41.4)	13 (14.9)	86 (56.6)	
Housewife/househusband	11 (4.6)	2 (2.3)	9 (5.9)	
In school/education/re-education	13 (5.4)	13 (15.0)	0 (0.0)	
Duration of untreated psychosis, mean (SD), years	1.2 (8.0)	1.4 (3.2)	1.0 (9.8)	0.7719^a^
Age at diagnosis, mean (SD), years	30.9 (13.0)	23.0 (4.3)	35.3 (14.2)	< 0.0001^a^
Time of diagnosis, n (%)				< 0.0001^b^
≤ 5 years	78 (32.4)	47 (53.4)	31 (20.3)	
> 5 years	163 (67.6)	41 (46.6)	122 (79.7)	
Number of illness episodes, n (%)				0.0002^b^
≤ 5 episodes	137 (57.1); 19 (7.9) of which with first episode of schizophrenia	64 (72.7)	73 (48.0)	
> 5 episodes	103 (42.9)	24 (27.3)	79 (52.0)	
BMI, mean (SD), kg/m^2^	29.3 (6.9)	28.6 (7.3)	29.7 (6.6)	0.2381^a^
BPRS at baseline, mean (SD)	54.1 (15.6) (*n* = 228, FAS)	53.0 (16.1) (*n* = 88, all values)	53.7 (15.9) (*n* = 151, all values)	0.7608^a^
CGI-S at baseline, mean (SD)	4.8 (0.8) (*n* = 235, FAS)	4.7 (0.9) (n = 89, all values)	4.8 (0.8) (n = 153, all values)	n/a
WHO-5 at baseline, mean (SD)	10.6 (5.6) (n = 235, FAS)	11.1 (5.7) (n = 88, all values)	10.5 (5.7) (*n* = 150, all values)	n/a
GAF at baseline, mean (SD)	47.0 (13.9) (n = 235, FAS)	49.9 (13.1) (n = 88, all values)	45.6 (14.6) (*n* = 152, all values)	n/a

^a^t-Test; ^b^ Fisher’s Exact Test; *BMI* Body Mass Index; *FAS* Full Analysis Set; *GAF* Global Assessment of Functioning; *WHO* World Health Organization; *SD* Standard deviation

Where percentages do not add up to 100%, data were missing for some patients. n/a: not available

## Results

Altogether, 278 patients were reported by physicians as potential study participants. 243 patients (87.4%) were included in the study cohort. One patient was excluded from the analysis because he did not receive any AOM injections. Patient baseline demographics and clinical characteristics are presented in Table 1. At the time of admission, the GAF score was 47.0 (±13.9), indicating that patients had on average serious symptoms or serious impairment in social, occupational, or school functioning. The patients reported a mean value of 10.6 (±5.6) on the WHO-5 wellbeing index. Thus, the patient’s wellbeing was below average (in the general population in Germany, scores of 15–18 are the norm) and that they were at risk of depression, considering a cutoff value of 13 [29]. Altogether 15 patients (6.2%) dropped out during the 6-month observation period and the mean study duration was 5.4 ± 1.0 months.

### Patient functioning (GAF)

**Fig. 2:**
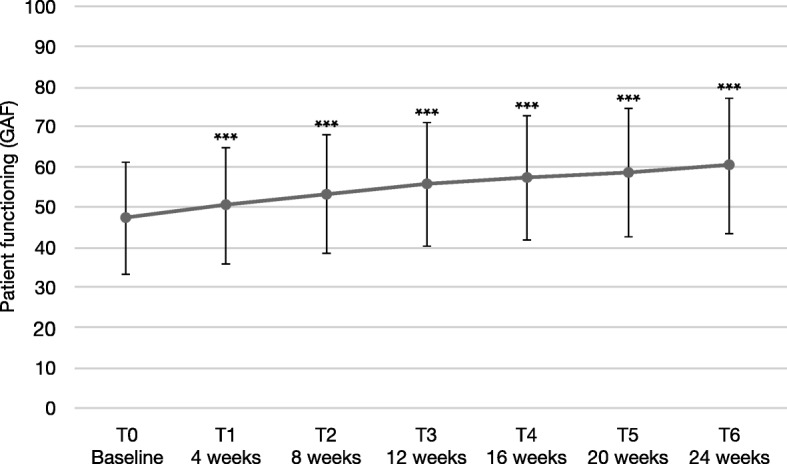
Patient functioning according to GAF scale. Error bars represent standard deviations. *** significant for each assessment versus baseline, *p* < 0.001

The overall patient functioning according to the GAF scale increased significantly during the observational period by 13.2 (SD 16.1), reaching 60.2 (±17.0) (Fig. 2). We found a continuous increase of values over treatment time. Altogether, 180 patients (76.6%) improved according to the GAF scale during the study, 35 (14.9%) remained unchanged, and 20 (8.5%) worsened. In total, 100 patients (42.6%) improved by ≥12 points, 130 (55.3%) improved by ≥10 points, and 166 (70.6%) by ≥4 points, representing possible criteria of clinically relevant changes [28].

**Fig. 3:**
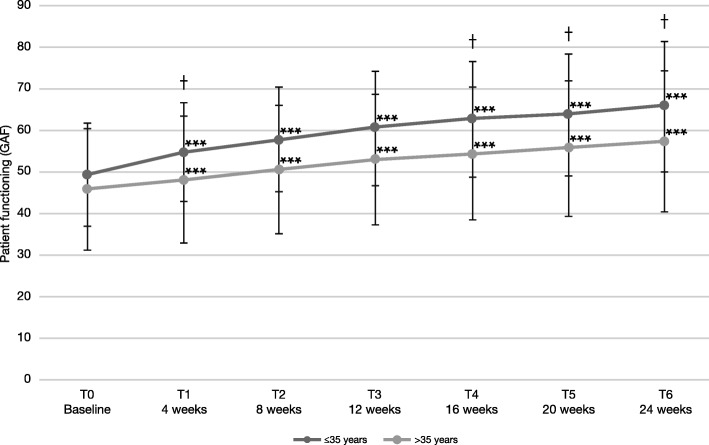
Patient functioning according to the GAF scale, stratified by age. Error bars represent standard deviations. At all visits, changes for both groups were significant compared to baseline (*** p < 0.001). Differences between groups were significant at T1, T4, T5, and T6; †, *p* < 0.05

In younger patients ≤35 years, the GAF score increased by 16.4 points (SD 18.3) on the GAF scale between T0 and T6. In contrast, patients > 35 years old increased only by 11.4 points (SD 14.4) (Fig. 3). The differences between the groups were statistically significant at T1, T4, T5 and T6 (Wilcoxon Two-Sample test, T1: *p* = 0.0092, T4: *p* = 0.0429, T5: *p* = 0.0459, T6: *p* = 0.0222). Moreover, 80.2% of the patients ≤35 years old improved during treatment, 12.8% remained unchanged, and 7.0% worsened. Regarding patients > 35 years old, 74.5% improved, 16.1% remained unchanged, and 9.4% worsened. Responders, defined as patients who improved by ≥4, ≥10, or ≥ 12 points, were included 77.9, 67.4%, or 54.7% of the younger vs. 66.4, 48.3%, or 35.6% of the older patients, respectively.

### Patient wellbeing (WHO-5)

**Fig. 4:**
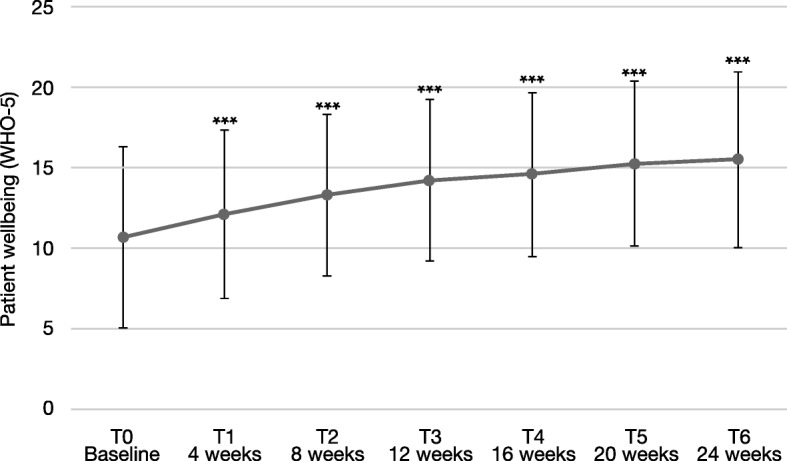
Patient wellbeing according to the WHO-5 wellbeing index. Error bars represent standard deviations. *** significant for each assessment versus baseline, p < 0.001

Patient wellbeing increased significantly by 4.8 points (SD 6.9) on the WHO-5 index (Fig. 4), reaching a mean of 15.4 ± 5.5 points at T6, a level that is comparable to the general population [29]. During the course of treatment, there was a continuous increase of the WHO-5 index values. The most prominent improvement was achieved during the first 4 weeks of the study. Altogether, 183 patients (77.9%) had improved wellbeing indices, 13 (5.5%) remained unchanged, and 39 (16.6%) had decreased wellbeing indices during the study.

**Fig. 5:**
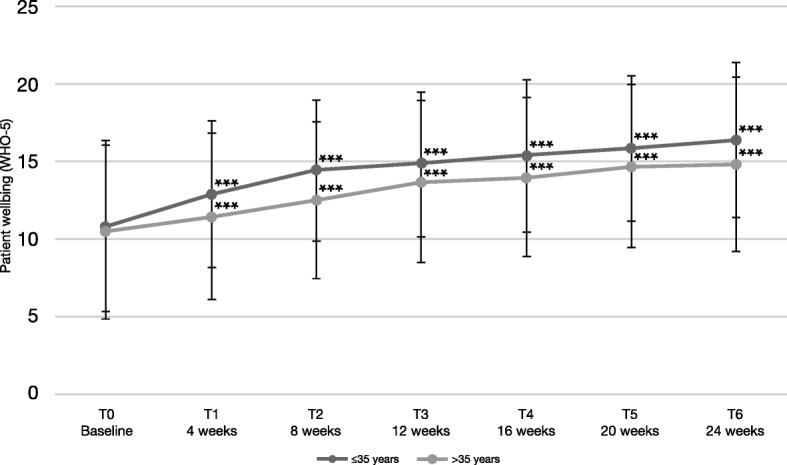
Patient wellbeing according to the WHO-5 wellbeing index, stratified by age. Error bars represent standard deviations. *** significant for each assessment versus baseline, *p* < 0.001. Between-group differences were not significant

In younger patients ≤35 years, there was an increase of 5.6 points in the wellbeing index (SD 7.4) between T0 and T6 (Fig. 5). In contrast, patients > 35 years old increased only by 4.4 points (SD 6.7). Furthermore, 81.4% of the younger patients had improved values at the end of the study, compared to 75.8% of the older patients. These differences were not statistically significant.

**Fig. 6:**
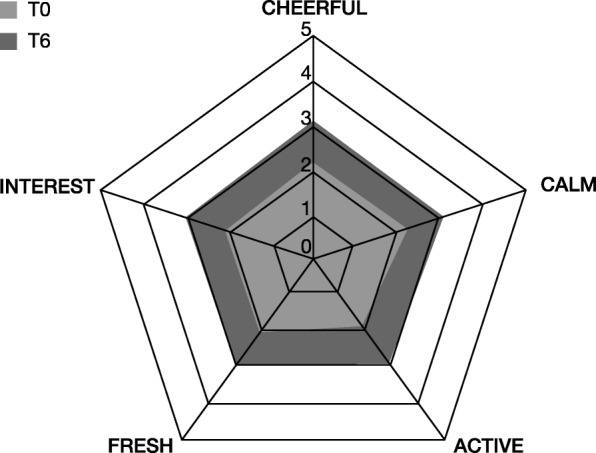
Individual items of patient wellbeing according to WHO-5 wellbeing index. Cheerful: “I have felt cheerful and in good spirits”, calm: “I have felt calm and relaxed”, active: “I have felt active and vigorous”, fresh: “I woke up feeling fresh and rested”, interest: “My daily life has been filled with things that interest me”

Regarding the individual items of the wellbeing index, the improvements across the items were similar (Fig. 6), with about a 1-point difference between baseline and follow up in each item. Again, there was a tendency for younger patients ≤35 years to experience greater improvements.

### Effectiveness and tolerability

**Fig. 7:**
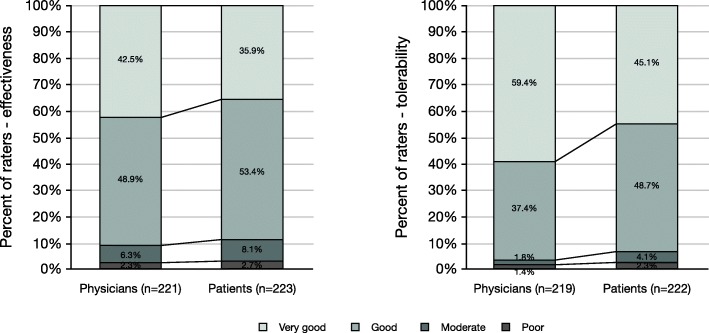
Assessment of effectiveness and tolerability of AOM treatment by treating physicians and patients

The effectiveness of AOM treatment was rated to be very good or good by 42.5% or 48.9% of the treating physicians, respectively (Fig. 7). The patients rated the effectiveness as very good in 35.9% and as good in 53.4% of the cases. Only 2.3% of the physicians and 2.7% of the patients gave a “poor” rating. Differences in patient and physician ratings of AOM effectiveness were not statistically significant.

The tolerability of AOM treatment was rated as very good by 59.4% of the treating physicians, 37.4% rated it as good. 45.1% of the patients rated AOM tolerability as very good and 48.7% as good. Only 1.4% of the physicians and 2.3% of the patients thought that AOM tolerability was poor. The differences in patient and physician ratings of AOM tolerability were statistically significant (*p* < 0.001).

### Reasons to switch to AOM treatment

Up to five reasons for switching the therapy to AOM were stated. The most frequent reasons reported by the treating physicians were assurance of compliance/adherence/drug intake (117, 48.4%), better/good tolerability (41, 16.9%), patient choice (33, 13.6%), better/good efficacy (29, 12.0%), and ease of administration (22, 9.1%, all based on total population).

### Continuation of therapy with AOM

After study completion, 200 patients (82.6%) continued their therapy with AOM, 24 (9.9%) discontinued it (data were missing for 18 patients). Reasons for discontinuation included patient choice (9, 3.7%), lack of efficacy (7, 2.9%), adverse drug reaction (6, 2.5%) and change to inpatient drug addiction therapy (1, 0.4%). One patient dropped out one patient moved away (0.4% each, all based on total population, one patient gave two reasons for discontinuation).

### Adverse events

Patients reported a total of 153 adverse events (AE) during the study, 133 of which were deemed as probably or possibly treatment related (TRAE) by the clinicians. All TRAEs affected less than 5% of the patients, except “medication taken at an inappropriate time” (*n* = 60, 24.8%). In these cases, oral aripiprazole was often stopped earlier than recommended (< 14 days) after AOM had been initiated. Common TRAEs as well as weight changes and extrapyramidal symptoms are reported in [27]. Only a few TRAEs occurred.

## Discussion

This non-interventional study was conducted to examine whether the positive effects of AOM treatment that have been previously reported in randomized controlled trials [15, 19, 20] could be replicated in and extended to a naturalistic setting with predominantly stable patients treated with oral aripiprazole before enrollment in the study [27]. Here, we report the outcomes of the GAF rating scale and the WHO-5 questionnaires that were used to assess patient functioning and wellbeing, as well as other outcomes that are relevant for the patient’s perspective on the treatment. The main findings were that both GAF and WHO-5 improved significantly and early in predominantly stable patients after switching from oral aripiprazole to AOM treatment.

At baseline, patients had a mean GAF value of 47.0 (SD: 13.9), indicating that they showed serious symptoms or serious impairment in social and/or occupational functioning. During treatment, the mean value continuously increased with statistically significant changes. Possible cutoff values to define a clinically relevant change have been discussed by Amri et al. [28]. Here, we report numbers for all three suggested thresholds (i.e., ≥4, ≥10, or ≥ 12 points). At T6, the mean GAF score was 60.2 (SD: 17.0), indicating that the serious symptoms or the difficulties in functioning that the patients had presented with at baseline had become more moderate. These results are consistent with results from a recent non-interventional study in stable Canadian patients treated with AOM that found an improvement from 49 to 61 GAF points within 1 year [31] and other previous findings [18, 32] as well as those of a post-marketing surveillance study of oral aripiprazole, where improvements of 15.8 points on the GAF scale were found [33], and of another naturalistic study that found a 14-point improvement within 6 months [34]. In two randomized controlled trials that studied the effects of AOM, previous improvements in functionality, assessed by the Personal and Social Performance (PSP) scale, could be maintained over long periods of 38–52 weeks [21]. Paliperidone palmitate LAI also allowed patients with schizophrenia to maintain their improvements on the PSP scale for 15 months [35]. In general, long-acting injectable formulations of second-generation antipsychotics seem to be effective in improving patient functioning and quality of life [36, 37].

Changes on the GAF scale are usually accompanied by respective changes on other symptomatic rating scales [38]. This relationship is consistent with our previously reported findings of improvements on BPRS and CGI-Scales in the same sample [27]. In an exploratory subgroup analysis, improvements on the GAF scale appeared more pronounced in younger patients, although this subgroup analysis had less statistical power. A shorter duration of psychotic illness and longer time of administration of a LAI was predictive of functional status in another study [39], which highlights that early use of LAI may improve outcome in those with schizophrenia-spectrum-disorders.

At baseline, the patients had a mean WHO-5 wellbeing index of 10.6 (±5.6), indicating that they were, on average, in a low mood. WHO-5 scores of ≤12.5 indicate low mood, while scores of ≤7 indicate likely depression [40]. In our study, patients improved significantly from 10.6 points to a mean of 15.4, which is comparable to the general population [29]. It is considered that a change of 10% or more on the scale is clinically relevant [29]. Taken together, all of this suggests that AOM treatment effectively improves patients` wellbeing. These data confirm the impressions recorded by the physicians via the GAF scale and suggest that improvement of patient functioning can lead to increased wellbeing. In general, previous studies found that patient’s subjective wellbeing and quality of life increase during antipsychotic treatment, often with superior results during atypical versus typical antipsychotic treatment [41–44].

Again, improvements on wellbeing were more pronounced in younger patients ≤35 years, yet, differences compared to patients > 35 years old were not statistically significant. An enhanced benefit of AOM treatment in patients aged ≤35 years was also apparent in the QUALIFY study, with marked improvements in the patients` quality of life [15] found in an exploratory, predefined subgroup analysis, which had, however, reduced statistical power. Early start of treatment may help to protect the patients from experiencing repeated episodes, which can cause long-term deterioration in psychosocial functioning and quality of life [2, 9, 45]. Patients, even after a first-episode of psychotic illness, show very high rates of non-adherence [9, 46]. This non-adherence poses a major risk factor for relapse or experiencing a further psychotic episode. When improving the subjective well-being of patients, higher adherence to medication could be expected [24–26], which can protect patients from having further psychotic episodes.

As antagonism of striatal and/or extrastriatal dopamine-D2 receptors can cause dysphoric experiences and depression [47–49], despite the high D2 occupancy of aripiprazole, the partial agonist profile at D2 receptors may possibly weaken this effect and can lead to a improved subjective wellbeing [50].

Although the ratings on positive therapeutic effects can differ between patients and physicians, in our study, both patients and physicians had similar ratings regarding the experienced or observed effectiveness of AOM.

Although the tolerability of AOM treatment was rated as “very good” or “good” by the majority of physicians and patients (96.8% vs. 93.7%), there were statistically relevant differences in ratings with physicians assuming a better tolerability than patients did. It seems that physicians may overestimate the tolerability of antipsychotic medication. Therefore, although in our study side effects were rare, this finding highlights the importance of asking patients very thoroughly if they are experiencing side effects, because adverse effects are one of the main reasons for non-adherence and treatment discontinuation [8, 51, 52].

The most important reason to switch to AOM treatment was that the adherence to treatment was assured. This result highlights on the one hand the importance of having long-acting injectables as treatment options for patients who have difficulties maintaining consistent drug intake, as maintenance of medication is one of the most important factors of staying relapse-free and relatively stable [4]. On the other hand, it should be emphasized that not only those with a history of non-adherence should be considered for treatment with an LAI, as non-adherence increases over time and as preventing relapses may be even more beneficial than targeting stability after another relapse has occurred [53]. In this study, we showed that even predominantly stable patients have the potential to improve further when switching from oral antipsychotic to LAI treatment. This is consistent with other mirror-image studies in which beneficial effects of LAI antipsychotics were found. Especially those in the first years of their illness will benefit the most from continuous treatment, which may prevent patients from the potential deteriorating effects of functional status and quality of life because of relapses and an ongoing illness process [4]. Furthermore, continuous long-term treatment seems to have a benefit regarding relapse-rates, treatment discontinuation as well as mortality [12, 54].

After the observational study period ended, most patients (*n* = 200, 82.6%) continued AOM treatment clinically, underscoring that they experienced the treatment as effective and tolerable and providing further face validity to this result. This finding is also consistent with a recent observational, retrospective, non-interventional study of 261 schizophrenia patients in which 86% of the patients continued using AOM for ≥6 months [55]. In our study, AOM was ineffective or elicited adverse reactions that led to discontinuation only in a small number of patients.

During the study, only a few TRAEs were recorded. All types of events had already been reported in other AOM studies. A more thorough discussion of TRAEs can be found in [27].

An important point of this study was to enroll a naturalistic patient population. Therefore, these predominantly stable patients were only enrolled after the treatment choice had already been made. During oral aripiprazole treatment, most patients had been symptomatically stable, as assessed by the treating psychiatrist (without use of a dedicated rating scale), for a mean duration of 5.9 months (SD 18.2), with 91 patients (39.2%) being stable for < 1 month and 28 (12.1%) being not stable at all. Patients in other clinical trials that investigated AOM effectiveness or safety showed similar demographic and epidemiologic data [19, 56–58]. These studies included between 59.4 and 77.4% of male patients, the mean patient age ranged from 40.1 to 45.2 years, and the mean BMI was between 28.1 and 28.7. The mean age of the first diagnosis ranging between 24.4 years [57] and 28.2 years [20] was also comparable to the one reported here. Therefore, the population analyzed here can be considered characteristic of patients suffering from schizophrenia.

Study limitations include the risk of selection bias (only patients willing to be treated with LAI and AOM medication), the lack of a control or comparator group, the relatively small sample of patients aged ≤35 years, reducing the power for subgroup analyses by age, and the risk of confounding factors due to non-randomization. Therefore, the results can just be interpreted as descriptive. The improvements seen could be due to the switch to the LAI, to improved adherence, or just due to longer time on an antipsychotic medication. Possibly, the same cohort would have improved similarly with an alternative treatment regimen (e.g. a different antipsychotic or even remaining on oral medication). Nevertheless, a recent meta-analysis of uncontrolled, open-label cohort studies suggested that LAI treatment is more efficacious than oral antipsychotic treatment for the prevention of hospitalization in people with schizophrenia [14]. The naturalistic, non-interventional design makes it impossible to identify or exclude possible confounders.

Although a shortcoming of the study is that no placebo group or parallel group was included, observational studies are an important complement to RCTs [59–61] due to their “real life” approach with an increased likelihood of a less biased inclusion of patients with psychotic disorders who might have denied study participation in a (randomized) controlled trial. The efficacy and safety of AOM has already been shown in controlled trials [19, 20, 57, 62]. The present study complements these data by observing a patient cohort that was not as strictly selected as a group for a randomized controlled trial. Therefore, patients with multiple co-morbidities, co-medications, and other risk factors were included. Our data show that AOM is also effective and safe in these patients.

## Conclusions

Our results support previous data collected in randomized controlled trials in a sample followed and treated under routine conditions in clinical practice. Taken together, our findings support that AOM is effective and safe for the outpatient treatment of patients with schizophrenia and that treatment with aripiprazole over time appears to have clinical benefits. Since we had no comparison group our findings have observational character and it can just be assumed why patients improve over the ensuing 6 months. The improvements could be related to a better adherence, more stable plasma levels of the medication and regular contacts with the treatment system.

Patient wellbeing and psychosocial functioning improved during AOM treatment under routine medical practice conditions. Both psychiatrists and patients found AOM treatment effective and tolerable. The positive therapeutic action seemed to be especially pronounced in younger patients, which underlines the need for early and continuous treatment to achieve these treatment outcomes that are important from both a clinician’s, family’s and, especially, the patient’s perspective.

## Data Availability

The dataset used and/or analyzed during the current study are available from the corresponding author on reasonable request.

## Abbreviations

AE: Adverse event
AOM: Aripiprazole once-monthly
BMI: Body Mass Index
BPRS: Brief Psychiatric Rating Scale
CGI-I: Clinical Global Impression – Improvement
CGI-S: Clinical Global Impression – Severity
GAF: Global Assessment of Functioning
ICD: International Classification of Diseases
LAI: Long-acting injectable
LOCF: Last observation carried forward
MedDRA: Medical Dictionary for Regulatory Activities
QUALIFY: Quality of Life with Abilify Maintena®
RCT: Randomized controlled trial
SD: Standard deviation
TRAE: Treatment related adverse event
WHO: World Health Organization
WHO-5: WHO-5 Well-Being Index
